# Genistein Binding to Copper(II)—Solvent Dependence and Effects on Radical Scavenging

**DOI:** 10.3390/molecules22101757

**Published:** 2017-10-18

**Authors:** Jing Yang, Yi Xu, Hao-Yu Liu, Rui-Min Han, Jian-Ping Zhang, Leif H. Skibsted

**Affiliations:** 1 Department of Chemistry, Renmin University of China, No. 59, ZhongGuanCun Street, Beijing 100872, China; yangjing2015@ruc.edu.cn (J.Y.); yixu@ruc.edu.cn (Y.X.); ruchyliu@gmail.com (H.-Y.L.); jpzhang@ruc.edu.cn (J.-P.Z.); 2 Department of Food Science, University of Copenhagen, Rolighedsvej 30, DK-1058 Frederiksberg C, Denmark

**Keywords:** genistein, copper, metal chelate, antioxidant

## Abstract

Genistein, but not daidzein, binds to copper(II) with a 1:2 stoichiometry in ethanol and with a 1:1 stoichiometry in methanol, indicating chelation by the 5-phenol and the 4-keto group of the isoflavonoid as demonstrated by the Jobs method and UV-visible absorption spectroscopy. In ethanol, the stability constants had the value 1.12 × 10^11^ L^2^∙mol^−2^ for the 1:2 complex and in methanol 6.0 × 10^5^ L∙mol^−1^ for the 1:1 complex at 25 °C. Binding was not detected in water, as confirmed by an upper limit for the 1:1 stability constant of K = 5 mol^−1^ L as calculated from the difference in solvation free energy of copper(II) between methanol and the more polar water. Solvent molecules compete with genistein as demonstrated in methanol where binding stoichiometry changes from 1:2 to 1:1 compared to ethanol and methanol/chloroform (7/3, *v*/*v*). Genistein binding to copper(II) increases the scavenging rate of the stable, neutral 2,2-diphenyl-1-picrylhydrazyl radical by more than a factor of four, while only small effects were seen for the short-lived but more oxidizing *β*-carotene radical cation using laser flash photolysis. The increased efficiency of coordinated genistein is concluded to depend on kinetic rather than on thermodynamic factors, as confirmed by the small change in reduction potential of −0.016 V detected by cyclic voltammetry upon binding of genistein to copper(II) in methanol/chloroform solutions.

## 1. Introduction

Polyphenols are a diverse group of phytochemicals acting as UV-light absorbers and singlet oxygen quenchers, protecting plant tissue against photodamage, and acting as chelators of ions of copper, iron, and other transition metals, thus protecting plant cells against oxidative degradation [1]. Among the plant phenols, the flavonoids are considered to be important micronutrients from fruit and vegetables. Benefits to human health of fruit and vegetables has been assigned to the anti-inflammatory, antiatherosclerotic, and antimutagenic activities of flavonoids. These and other positive effects of a high intake of flavonoids from fruit and vegetables on human health have often been assigned to radical scavenging capabilities [1,2,3,4]. The detailed mechanism behind such antioxidative protection seems, however, unclear, as most flavonoids under some conditions also act as prooxidants [5,6].

The balance between prooxidative and antioxidative properties of flavonoids seems to be related to the presence of ions of transition metals. Transition metal ions are redox active and may be active in generating radicals through cleavage of peroxides after reduction to the lower oxidation states of the metal ion by compounds like the flavonoids [7,8,9]. On the other hand, flavonoids also chelate transition metal ions, modifying both the redox properties of the flavonoid and of the metal ion. Metal flavonoid complexes have many biological activities, i.e., as antioxidants, and as inflammatory, anticancer, and antiallergic agents [10,11,12]. The interaction of flavonoids and metal ions is only poorly understood. The metal binding is strongly dependent on the solvent and is labile, thus hampering characterization of metal-flavonoid compounds [13], and the crystal structure is only known for a few metal-flavonoid complexes [14,15,16]. Experimental data related to antioxidant activities are often controversial. Some results indicate metal-flavonoid complexes exhibit higher antioxidant activity than their parent flavonoids [13,17,18,19,20,21]. However, in some cases, a reduction in biological activity after complexation by metals has been observed [22,23,24,25,26].

**Scheme 1:**
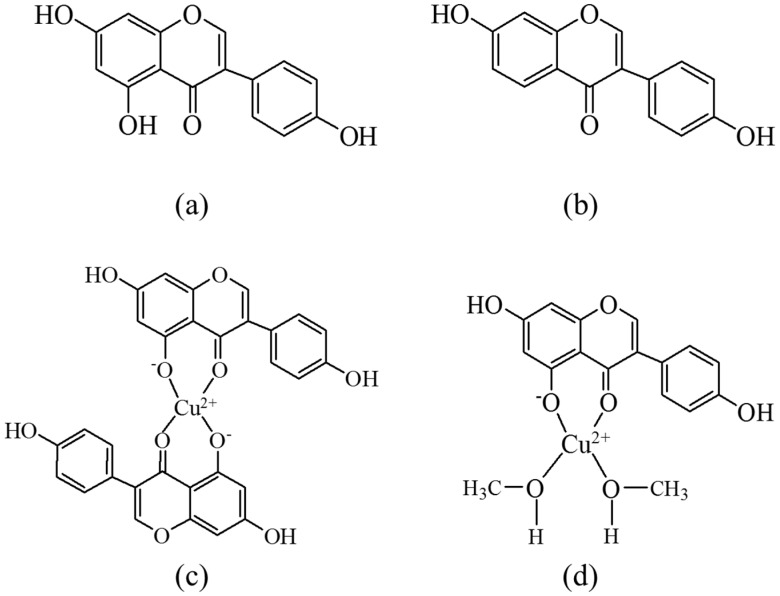
Molecular structures of (**a**) genistein; (**b**) daidzein, and proposed structures of copper(II)-genistein complexes as formed in (**c**) methanol/chloroform (7/3, *v*/*v*) and ethanol, and in (**d**) methanol.

Genistein (Scheme 1) is an isoflavonoid found in soybeans, fava beans, and other important stable foods for which chelation of copper(II) was studied using spectroscopic, time resolved photolytic methods and electrochemical methods in order to understand the modification of this important isoflavonoid as an antioxidant through metal chelation. In order to identify the functional groups of isoflavonoids crucial for chelation, the complex formation of genistein was compared with complex formation of the structurally similar daidzein, also shown in Scheme 1. The study of genistein chelation holds the perspective for design of improved antioxidant protective systems and of drug delivery systems.

## 2. Results

**Figure 1:**
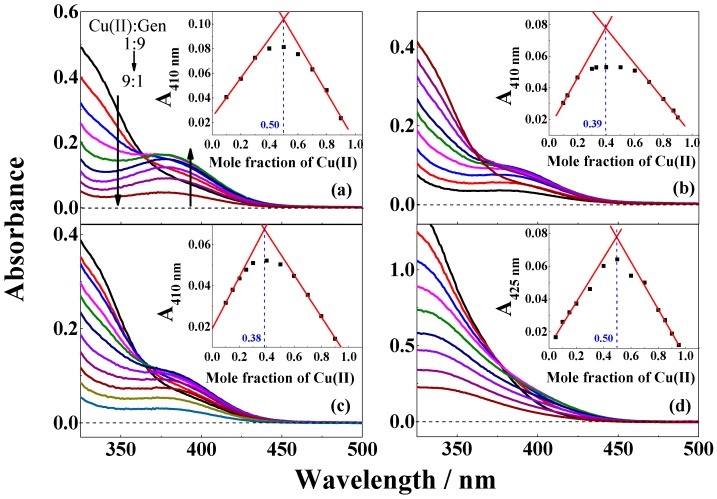
Absorption spectra of genistein and CuSO_4_ in different ratios varying from 9:1 to 1:9 in (**a**) methanol; (**b**) ethanol; (**c**) methanol/chloroform (7/3, *v*/*v*); and (**d**) in the presence of 2 equivalent (CH_3_)_4_N^+^OH^‒^ relative to concentration of genistein in methanol/chloroform (7/3, *v*/*v*). The inserts in figures are the corresponding Job’s plots for the absorbance (**a**–**c**) at 410 nm and (**d**) at 425 nm versus mole fraction of CuSO_4_. The total concentration of genistein/genistein^2^^‒^ and CuSO_4_ is 100 µM.

**Figure 2:**
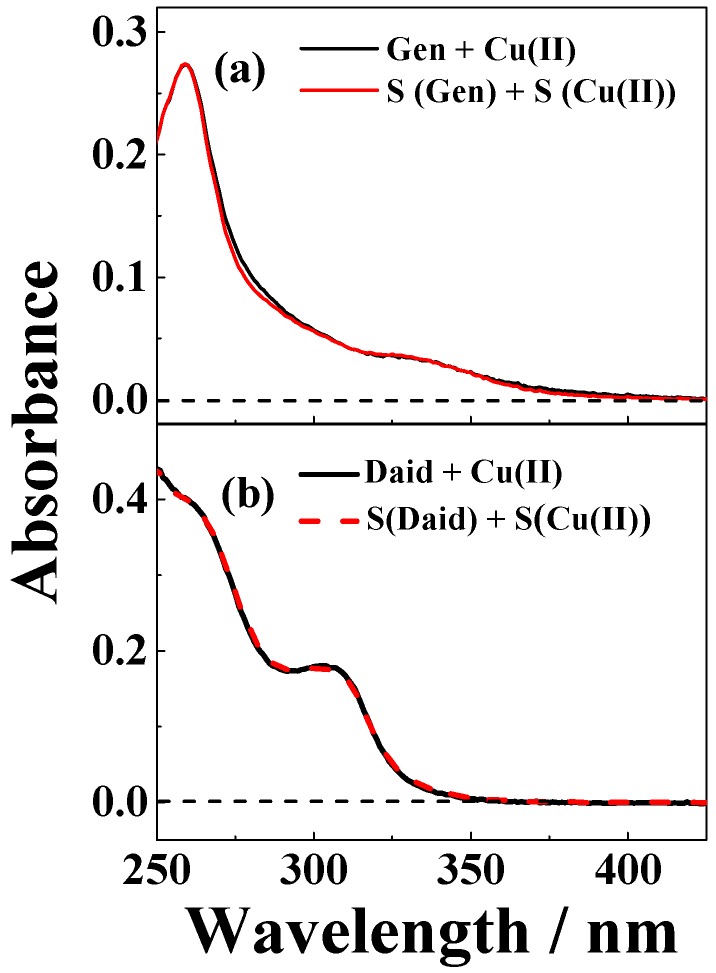
Absorption spectra of a solution of (**a**) 25 μM genistein and 25 µM CuSO_4_, and the superposition spectra S(Gen) + S(Cu(II)) of 25 µM genistein and 25 µM CuSO_4_ in water with 1% ethanol solution; and (**b**) 20 µM daidzein and 20 µM CuSO_4_, and the superposition spectra S(Daid) + S(Cu(II)) of 20 µM daidzein and 20 µM CuSO_4_ in methanol/chloroform (7/3, *v*/*v*).

The UV-visble absorption spectra of copper(II) sulfate dissolved in ethanol, methanol, or in methanol/chloroform (7/3, *v*/*v*) change upon additon of the isoflavonoid genistein, as may be seen from Figure 1a,c. The spectral changes observed were assigned to coordination of genistein to copper(II). The genistein absorption in neutral methanol/chloroform (7/3, *v*/*v*) consists of an absorption band with a maximum at 262 nm and an absorption shoulder occuring between 320–400 nm (Figure 1 and Figure 2). Upon addition of copper(II), the absorbance band broadens, while the absorbance shoulder shows a significant bathochromic shift to 425 nm. Similar changes were not seen for copper sulfate dissolved in water (Figure 2a) or in tetrahydrofuran (THF, data not shown) with addition of genistein.

The chelating properties of genistein was supported by the spectral changes observed for copper(II) in the presence of genistein but not of daidzein (Figure 2b). Based on the structures of genistein and daidzein, as are shown in Scheme 1, it was concluded that the 5-hydroxy group present in genistein, but not in daidzein, is important for complex formation for copper(II) with isoflavonoids.

The stoichiometry of the complex formation of copper(II) by genistein in the different solvents was investigated using Job’s method of continous variation [27]. Absorbance at 410 nm for mixtures of copper(II) and genistein in different ratios varying from 9:1 to 1:9 in methanol, ethanol, and methanol/chloroform (7/3, *v*/*v*) showed an absorption maximum identified by a duplicate linear regression, as is seen from the inserts in Figure 1, which correspond to compositions of the complexes. For methanol/chloroform (7/3, *v*/*v*) under basic conditions with (CH_3_)_4_N^+^OH^−^ added, absorbance at 425 nm was used to identify the composition of the complex. In ethanol, both under neutral and basic conditions and methanol/chloroform (7/3, *v*/*v*), a ratio of 1:2 between copper(II) and genistein was determined, while in methanol both under neutral and basic conditions and in methanol/chloroform (7/3, *v*/*v*) under basic condition, a 1:1 ratio became evident, as seen in Figure 1 and Table 1. Similar 1:1 and 1:2 complex formation between copper(II) and genistein was also reported by Dowling et al. in methanol at different pH [13]. The proposed structures for the 1:2 complex formed in ethanol and in methanol/chloroform (7/3, *v*/*v*) and for the 1:1 complex formed in methanol are shown in Scheme 1. As will be discussed below, these structures further draw support from the mass spectra of the complexes formed under the different conditions.

The stability constants for the 1:1 and 1:2 complexes formed under the different solvent conditions

(1) $${\mathrm{Cu}}^{2+}+\mathrm{Gen}⇌\mathrm{Cu}{\left(\mathrm{Gen}-H\right)}^{+}+H^{+}$$

(2) $${\mathrm{Cu}}^{2+}+2\mathrm{Gen}⇌\mathrm{Cu}{\left(\mathrm{Gen}-H\right)}_{2}+{2H}^{+}$$

were determined using the relationship (3) according to the method of reference [30] for the 1:1 complex formation corresponding to the equilibrium of Equation (1),

(3) $$A_{e}/{\left[M_{0}\right]}^{\frac{1}{2}}=\varepsilon_{C}{\left[M_{0}\right]}^{\frac{1}{2}}+\left(\varepsilon_{G}-\varepsilon_{C}\right)/{K_{C}}^{\frac{1}{2}}$$

and using the relationship (4) for the 1:2 complex formation corresponding to the equilibrium of Equation (2),

(4) $$A_{e}/{\left[M_{0}\right]}^{\frac{1}{3}}=\varepsilon_{0}{\left[M_{0}\right]}^{\frac{2}{3}}+[1/{\left({4K}_{C}\right)}^{\frac{1}{3}}]\left({2\varepsilon }_{G}-\varepsilon_{C}\right)$$

**Figure 3:**
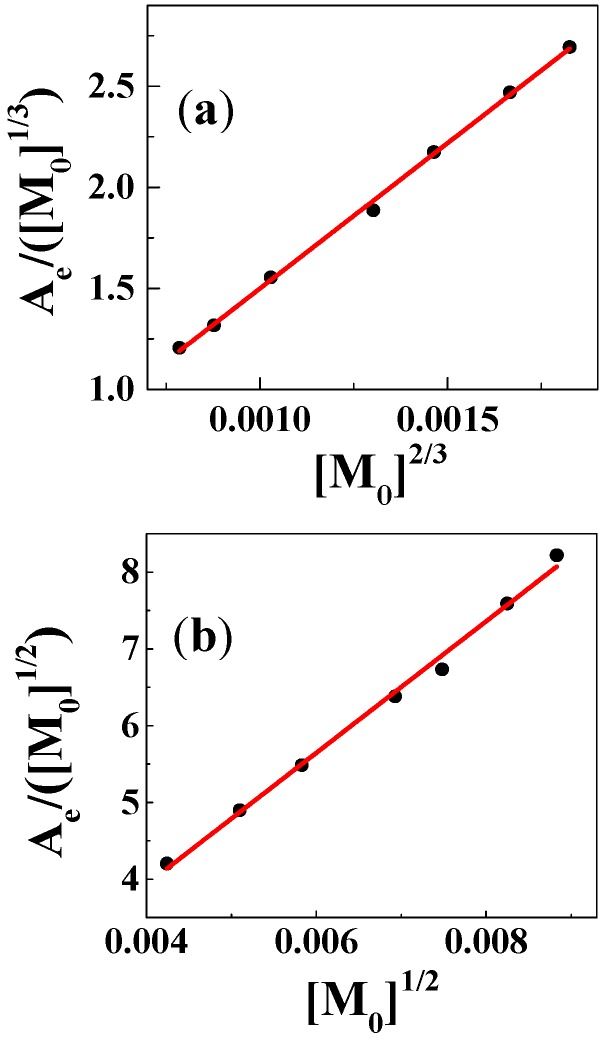
Determination of stability constants and molar absorptivity of the complexes using concentrations of CuSO_4_ ranging from 18 μM to 78 μM for (**a**) CuSO_4_:genistein = 1:2, *λ* = 410 nm, in methanol/chloroform (7/3, *v*/*v*) and for (**b**) CuSO_4_:genistein = 1:1, *λ* = 415 nm, in methanol.

In Equations (3) and (4), [M_0_] is the initial concentration of copper(II), while **ε**_G_ and **ε**_C_ are the molar absorptivity of genistein and the complex, respectively. For neutral methanol/chloroform (7/3, *v*/*v*) with formation of the 1:2 complex, the solution with copper(II) concentrations ranging from 18 μM to 78 μM and genistein concentrations ranging from 36 μM to 156 μM, absorbance at 410 nm formed the basis for determination of a stability constant of 1.1 × 10^11^ L^2^∙mol^−2^ valid for 25 °C based on Equation (4) as seen in Figure 3. For the 1:1 complex formed in methanol, using Equation (3), a stability constant of 6.0 × 10^5^ L∙mol^−1^ was determined for 25 °C based on Equation (3). The stability constants determined using this graphical method are collected in Table 1 for the three solvents methanol, ethanol, and the methanol/chloroform (7/3, *v*/*v*) for neutral and basic conditions. The stability constant 1.1 × 10^11^ L^2^∙mol^−2^ for 1:2 complex in ethanol is comparable to most 1:2 flavonoid-metal complexes from the literature, such as the rutin copper(II) complex in ethanol/water (5.7 × 10^10^ L^2^∙mol^−2^), 3-hydroxyflavone aluminium(III) complex in methanol (1.6 × 10^11^ L^2^∙mol^−2^), and the quercetin iron(II) complex in phosphate buffer (5.0 × 10^10^ L^2^∙mol^−2^). However, the stability constant 6.0 × 10^5^ L∙mol^−1^ for 1:1 complex in methanol is higher than of the results in the literature, such as the kaempferol iron(III) complex in acetate buffer (2.2 × 10^3^ L∙mol^−1^), myricetin copper(II) complex in ethanol/water (9.5 × 10^3^~1.9 × 10^3^ L∙mol^−1^), and the morin barium(II) complex in ethanol/water (3.5 × 10^4^ L∙mol^−1^) [10]. The polarity (P) of the three solvents and of water and THF, for which no complex formation was detected, are also available in Table 1 for comparison for the neutral solutions based on the dielectric constants (ε).

**Figure 4:**
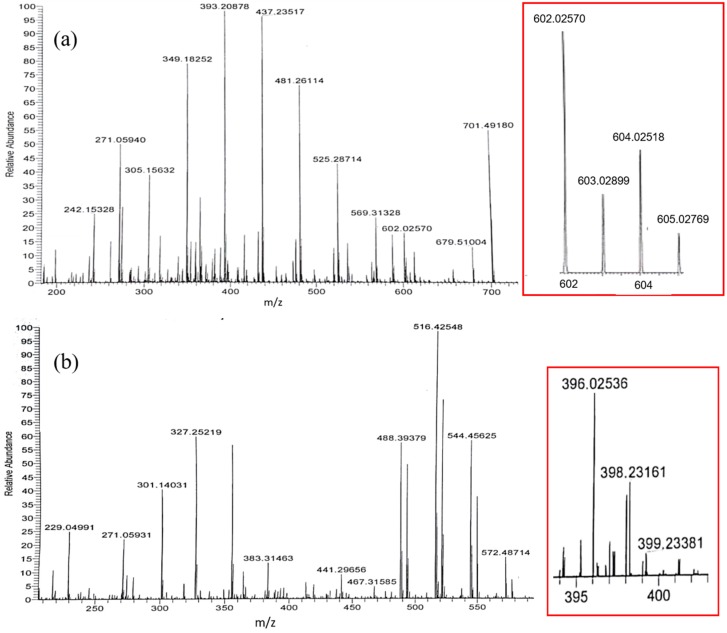
ESI-mass spectra of the mixed solution and of (**a**) 100 μM genistein and 50 μM CuSO_4_ in methanol/chloroform (7/3, *v*/*v*); and of (**b**) 100 μM genistein and 100 μM CuSO_4_ in methanol. (Right frame: Enlarged copper isotopic peaks of copper genistein complexes).

Mass spectrometry was used for determining the molecular weight of the copper(II)-genistein complex as formed, and the results for methanol and methanol/chloroform (7/3, *v*/*v*) are shown in Figure 4 and in Table 2. The signal at *m*/*z* 602 was assigned to Cu(II) + (Gen‒H)_2_ + H^+^ for the 1:2 complex in methanol/chloroform (7/3, *v*/*v*), and for methanol as solvent, the signal at *m*/*z* 396 was assigned to Cu(II) + (Gen‒H) + 2CH_3_OH for 1:1 complex, as shown in Scheme 1, in agreement with the results obtained by the Jobs method.

**Figure 5:**
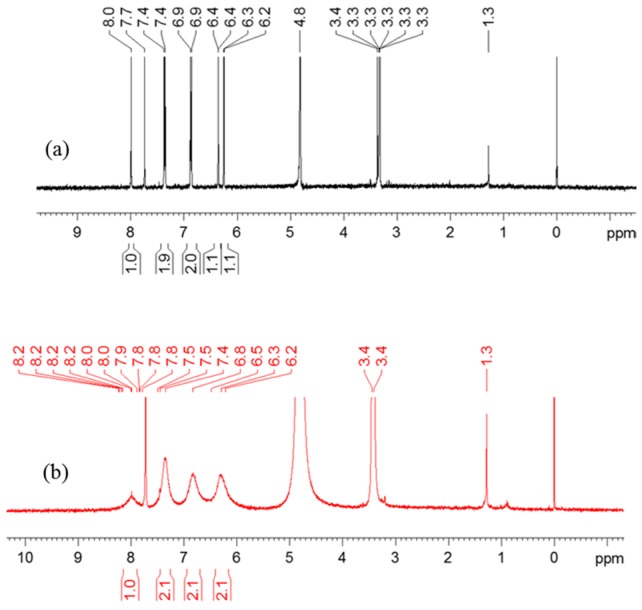
^1^H-NMR spectra of (**a**) 2 mM genistein and (**b**) the mixed solution of 16 mM genistein and 8 mM CuSO_4_ in methanol/chloroform (7/3, *v*/*v*).

^1^H-NMR spectra of genistein and copper(II)-genistein complex were obtained using methanol/chloroform (7/3, *v*/*v*) as solvent. The chemical shifts of each compounds as may be seen in Figure 5 are listed in Table 3. ^1^H-NMR data shows the values of the chemical shifts δ of the CuGen_2_ with characteristic broadened signals of the copper(II) complex changed to lower field as compared to the genistein [31]. Chemical shifts are transferred to lower field due to the increase of the conjugation caused by coordination of copper(II) to genistein, which increases the planarity of the flavonoid molecule [32]. The peaks are broadened, as copper(II) is paramagnetic with a d^9^ electronic configuration, and copper(II) added to genistein accordingly will disturb the magnetic field.

**Figure 6:**
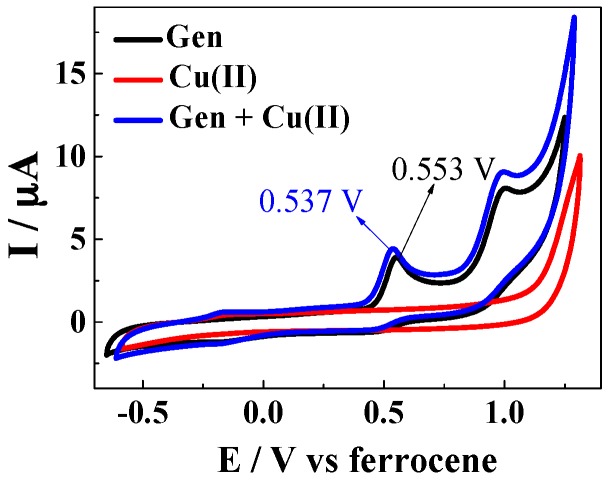
Cyclic voltammograms of a solution of 100 μM genistein, a solution of 50 μM CuSO_4_, and a solution of 100 μM genistein and 50 μM CuSO_4_ obtained in methanol/chloroform = 7/3 solutions with 0.10 M NaClO_4_ and 50 μM ferrocene.

The standard reduction potential of the copper(II) complex with genistein was determined in the methanol/chloroform (7/3, *v*/*v*) in which the 1:2 complex is formed and compared to the standard reduction potential of genistein and of copper(II) using cyclic voltametry. The cyclic voltammograms of genistein and copper(II) complex show characteristic oxidation peaks of flavonoids and no reversible reduction peaks, which were generally ascribed to two electron electrochemical process [33,34]. The cyclic voltammograms for all three compounds for 25 °C are shown in Figure 6, from which half wave potentials of 0.553 V for genistein and 0.537 V for Cu(Gen)_2_ were obtained.

Genistein and the copper(II)-genistein complexes formed were compared as antioxidants using two methods for determination of radical scavenging, one based on the very reactive carotenoid radical cations and the other based on a semi-stable slow reacting radical. Polyphenols are known as scavengers of carotenoids radical cations (Car^•+^), which are the first degradation product of carotenoids under oxidative stress:

(5) $$\beta \text{-}\mathrm{Car}\to {\beta \text{-}\mathrm{Car}}^{•+}+e^{-}$$

**Figure 7:**
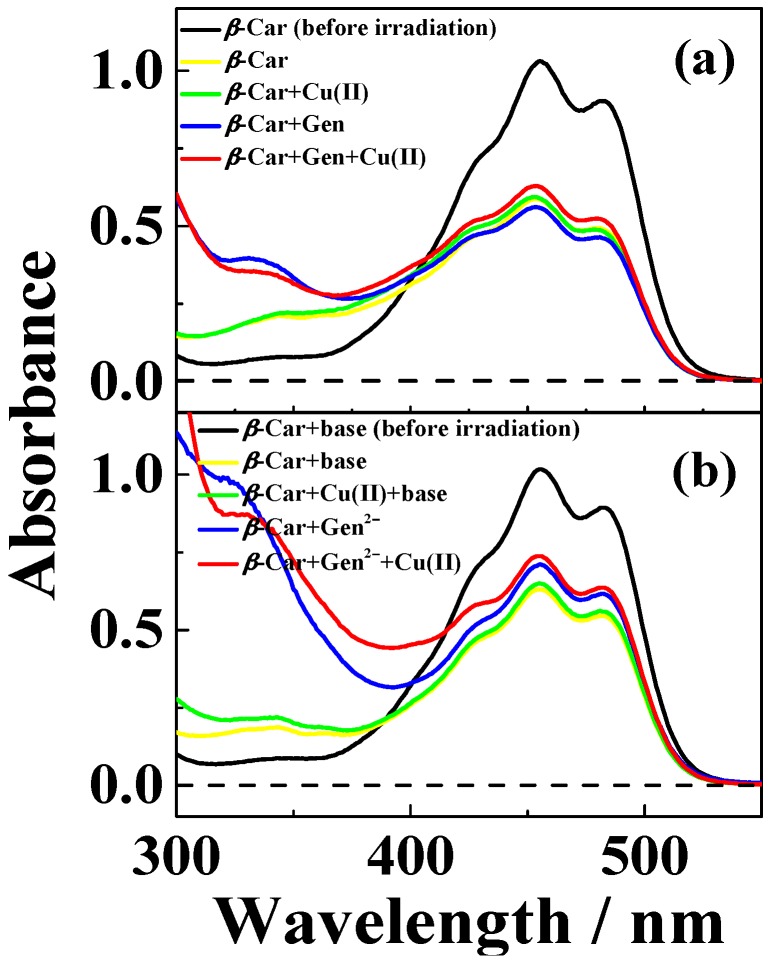
Absorption spectra of solutions of CuSO_4_, genistein and *β*-Car methanol/chloroform (7/3, *v*/*v*) before and after laser flash photolysis at 532 nm for (**a**) neutral and (**b**) basic conditions (addition of 100 µM (CH_3_)_4_N^+^OH^‒^). (**a**) 7 μM *β*-Car prior to laser flash irradiation (**―**), 7 μM *β*-Car and 50 μM genistein after 3 min irradiation (**―**), 7 μM *β*-Car and 25 μM CuSO_4_ after 3 min irradiation (**―**) and 7 μM *β*-Car, 50 μM genistein and 25 μM CuSO_4_ after 3 min irradiation (**―**); (**b**) 7 μM *β*-Car prior to laser flash irradiation (**―**), 7 μM *β*-Car and 50 μM genistein^2^^‒^ after 3 min irradiation (**―**), 7 μM *β*-Car and 50 μM CuSO_4_ after 3 min irradiation (**―**) and 7 μM *β*-Car, 50 μM genistein^2^^‒^ and 50 μM CuSO_4_ after 3 min irradiation (**―**). 50 µM genistein^2^^‒^ was formed from addition of 100 µM (CH_3_)_4_N^+^OH^‒^ to 50 µM neutral genistein solution.

Bleaching of *β*-Car upon irradiation with monochromatic 532 nm light was protected moderately by the presence of the 1:2 copper(II)-genistein complex in neutral methanol/chloroform (7/3, *v*/*v*), whereas genistein increased bleaching, and copper(II) had no effect, as is evident from Figure 7a. For basic conditions, the protective effect against bleaching of the 1:1 copper(II)-genistein complex is more significant, and genistein also shows some effect, while copper(II) has no effect, see Figure 7b.

**Figure 8:**
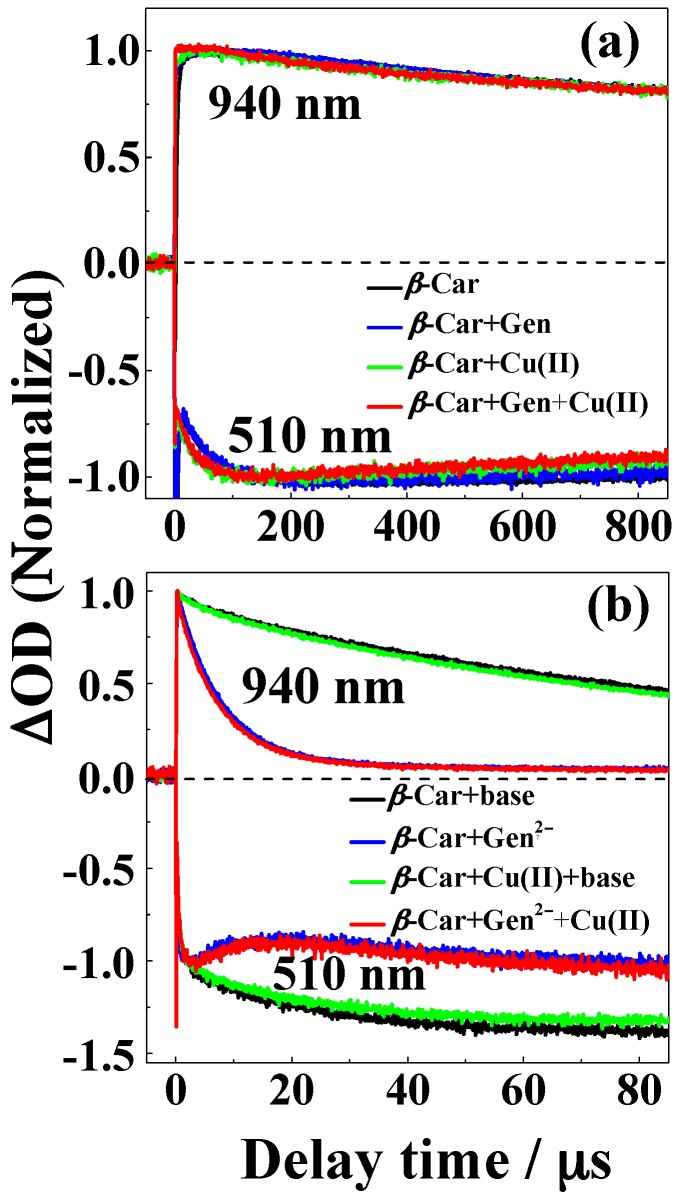
Normalized time evolutions of absorbance at 940 and 510 nm following pulsed photoexcitation using laser flash photolysis with 7 ns flash for (**a**) neutral and (**b**) basic solutions. (**a**) For a solution of 40 μM *β*-Car, a solution of 40 μM *β*-Car and 80 μM genistein, a solution of 40 μM *β*-Car and 40 μM CuSO_4_, and a solution of 40 μM *β*-Car, 80 μM genistein and 40 μM CuSO_4_; (**b**) For a solution of 40 μM *β*-Car, a solution of 40 μM *β*-Car and 40 μM genistein^2‒^, a solution of 40 μM *β*-Car and 40 μM CuSO_4_, and a solution of 40 μM *β*-Car and 40 μM genistein^2‒^ and 40 μM CuSO_4_ in methanol/chloroform (7/3, *v*/*v*). 40 μM genistein^2‒^ was formed from addition of 80 µM (CH_3_)_4_N^+^OH^‒^ to 40 μM neutral genistein solution.

The moderate protection of *β*-Car against bleaching was studied in more details using time resolved absorption spectroscopy (Figure 8). Bleaching of *β*-Car in neutral methanol/chloroform (7/3, *v*/*v*) was found to occur on the μs time scale, and the 1:2 copper(II)-genistein complex showed only little protection in agreement with the steady state absorption results. For basic conditions, a more clear protection became evident, as both genistein and the 1:1 copper(II)-genistein complex efficiently scavenged the *β*-Car^•^^+^ reproducing *β*-Car with no or very little difference between genistein and the 1:1 copper(II)-genistein complex. Scavenging of the fast reacting *β*-Car radical cation was confirmed for genistein by time resolved absorption at 940 nm where *β*-Car^•^^+^ is known to absorb, but complex formation showed little if any improvement of this antioxidative activity.

**Figure 9:**
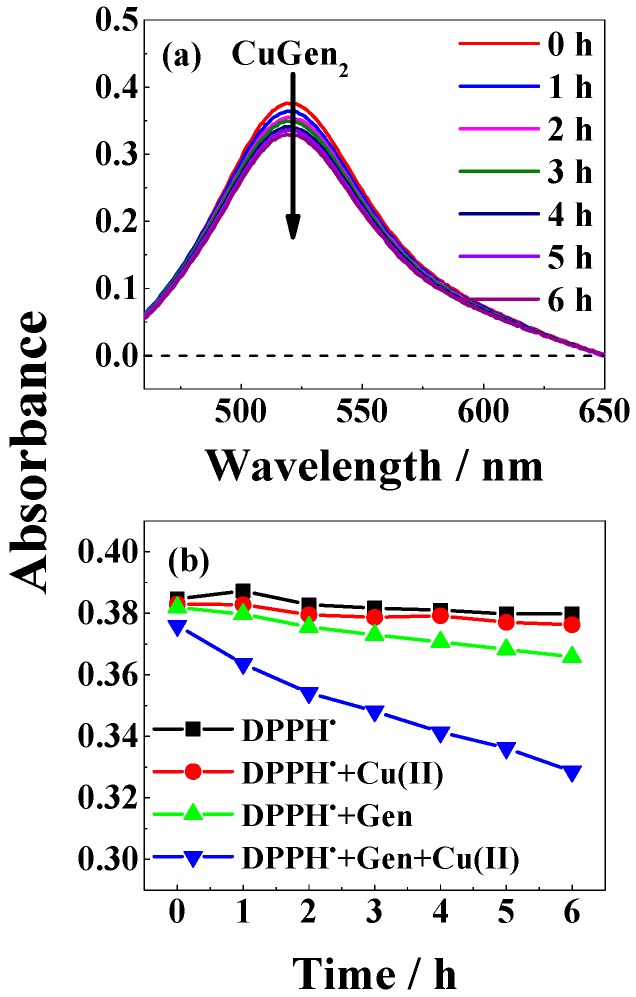
(**a**) Absorption spectra of 35 μM DPPH^•^ in the presence of 100 μM genistein and 50 μM CuSO_4_ in methanol/chloroform (7/3, *v*/*v*) at indicated delay times; (**b**) Time profile for bleaching of DPPH^•^ and of DPPH^•^ by 100 μM genistein, by 50 μM CuSO_4_, and by 100 μM genistein and 50 μM CuSO_4_ followed by absorbance at 521 nm for methanol/chloroform (7/3, *v*/*v*) as solvent.

For the slow reacting 2,2-diphenyl-1-picrylhydrazyl radical (DPPH^•^) in neutral methanol/chloroform (7/3, *v*/*v*), a clearer effect of the copper(II)-genistein complex became evident as an example followed by absorption spectroscopy (Figure 9a). Copper(II) was not found to react with DPPH^•^ resulting from the time profile in Figure 9b. Genistein had some scavenging effect, but clearly complex binding of genistein to copper(II) increased the scavenging rate by a factor of approximately four. This method was not explored under basic conditions like *β*-Car bleaching experiments due to instability of DPPH^•^ under such conditions.

## 3. Discussion

Cu(II) is a d^9^ system and is subject to Jahn-Teller effects. The solvated copper ion is a distorted octahedron with the four planar ligands of equal bond length, while the two axial ligands have a longer bond length as has been demonstrated for water as solvent [35]. The four equatorial water ligands are more strongly bonded as is reflected by p*K*_a_ = 6.5 [36] for the copper(II) aqua ion

(6) $$\mathrm{Cu}{{\left(H_{2}O\right)}_{4}}^{2+}⇌\mathrm{Cu}{\left(H_{2}O\right)}_{3}{\mathrm{OH}}^{+}+H^{+}$$

Cu(II) is bound more weakly in solvents less polar than in water. The free energy of copper(II) transfer from water to methanol corresponding to the reaction (7)

(7) $${\mathrm{Cu}{\left(H_{2}O\right)}_{4}}^{2+}+{4\mathrm{CH}}_{3}\mathrm{OH}\to \mathrm{Cu}{{\left({\mathrm{CH}}_{3}\mathrm{OH}\right)}_{4}}^{2+}+{4H}_{2}O$$

has the value $\Delta G^{\theta }=28.9\;\mathrm{kJ}\cdot {\mathrm{mol}}^{-1}$ as determined electrochemically [37], while the value for transfer of copper(II) from water to ethanol corresponding to the reaction (8)

(8) $${\mathrm{Cu}{\left(H_{2}O\right)}_{4}}^{2+}+{4\mathrm{CH}}_{3}{\mathrm{CH}}_{2}\mathrm{OH}\to {\mathrm{Cu}{\left({\mathrm{CH}}_{3}{\mathrm{CH}}_{2}\mathrm{OH}\right)}_{4}}^{2+}+{4H}_{2}O$$

has $\Delta G^{\theta }=50.6\;\mathrm{kJ}\cdot {\mathrm{mol}}^{-1}$ indicating that ethanol is more weakly bound to copper(II) than methanol. From the stability constant determined for the 1:1 copper(II)-genistein complex in methanol, see Table 1, $K_{C}=6.0\times 10^{5}\;{\mathrm{mol}}^{-1}\cdot L$, a value of $\Delta G^{\theta }=-33.0\;\mathrm{kJ}\cdot {\mathrm{mol}}^{-1}$ may be calculated for

(9) $${\mathrm{Cu}{\left({\mathrm{CH}}_{3}\mathrm{OH}\right)}_{4}}^{2+}+\mathrm{Gen}\to \mathrm{Cu}{\left({\mathrm{CH}}_{3}\mathrm{OH}\right)}_{2}{\mathrm{Gen}}^{2+}+{2\mathrm{CH}}_{3}\mathrm{OH}$$

which combined with the reaction of Equation (7) for the complex formation in water

(10) $${\mathrm{Cu}{\left(H_{2}O\right)}_{4}}^{2+}+\mathrm{Gen}\to \mathrm{Cu}{\left(H_{2}O\right)}_{2}{\mathrm{Gen}}^{2+}+{2H}_{2}O$$

gives a value of $\Delta G^{\theta }=-4.1\;\mathrm{kJ}\cdot {\mathrm{mol}}^{-1}$ corresponding to an equilibrium constant of $K_{C}={\mathrm{mol}}^{-1}\cdot L$ for the coordination of one genistein as ligand to copper(II) in water at 25 °C. The strong binding of water compared to methanol explains the observation, that complex formation between genistein and copper(II) in water could not be detected spectrophotometrically, since the equilibrium of Equation (10) with K_C_ = 5 ${\mathrm{mol}}^{-1}\cdot L$ will only entail 10^−2^% complex formation for the concentrations of 25 × 10^−6^ mol∙L^−1^ for both copper(II) and genistein as used in the present investigation. A similar calculation based on a comparison between complex formation in water and ethanol and $\Delta G^{\theta }$ for the reaction of Equation (8) gives a comparable result. The strong solvation of copper(II) salts in water accordingly prevents genistein as a relatively weak ligand of coordination to copper in this strongly polar solvent. Methanol and especially ethanol solvate copper(II) more weakly, resulting in a 1:1 complex formation in the more polar of the two alcohols, methanol, and a 1:2 complex in ethanol. The tendency of genistein to bind to copper(II) seems to be inversely correlated with the polarity of the solvent, see Table 1. Genistein was, however, not found to bind to copper(II) in THF, and other effects may be important for this aprotic solvent including a stronger solvation of the more hydrophobic genistein by THF.

Genistein is known as a reductant and a radical scavenger and both functions are important for the antioxidative activity of genistein. The standard reduction potential only changed from 0.553 V to 0.537 V by binding to copper(II). A similarly minor difference was also found for quercetin (0.62 V) and quercetin copper(II)/iron(II) complexes (0.58 V), and for 3-hydroxyflavone (0.91 V) and the 3-hydroxyflavone copper(II) complex (0.89 V) [33]. In contrast, the radical scavenging efficiency of genistein was found to increase in reaction with the slow reaction radical DPPH^•^ upon complex binding. The radical scavenging effect was, in contrast, not enhanced in the reaction with the fast reacting carotenoid radical cation *β*-Car^•^^+^ suggesting different mechanisms behind the radical scavenging.

Two molecular mechanisms behind the increased rate of radical scavenging of DPPH^•^ by genistein upon binding to copper(II) need to be considered. One mechanism would entail reduction of copper(II) to copper(I) by genistein

(11) $${\mathrm{Cu}}^{2+}+\mathrm{Gen}\to {\mathrm{CuGen}}^{2+}\to {\mathrm{Cu}}^{+}+{\mathrm{Gen}}^{•+}$$

followed by a reaction of the copper(I) formed with DPPH^•^. The equilibrium concentration of copper(I) may be calculated from the standard reduction potentials for copper(II) and for genistein. The equilibrium constant for the overall reaction of Equation (11) has a value of approximately 10^−10^, as calculated from the standard reduction potentials, and for the concentration intervals of interest, an equilibrium concentration of around 10^−10^ M for copper(I) is estimated. This concentration seems too low to be identified as the actual reactant for reacting with DPPH^•^. For the actual concentrations, bimolecular scavenging of DPPH^•^ by copper(I) would have to be faster than the diffusion limit in order to be significant.

Another molecular mechanism depends on perturbation of the conjugated bonding systems of genistein upon coordination to copper(II). A clearer understanding of such effects will depend on theoretical calculations of the electron distribution in genistein and genistein coordinated to copper(II). The difference between the radical scavenging efficiency of the copper(II)-genistein complexes observed for the *β*-Car radical cation and the DPPH^•^ may result from a difference in radical scavenging mechanism. *β*-Car^•^^+^ cannot accept a hydrogen atom and is rather scavenged by electron transfer [38], which may also explain the minor difference in the oxidation potentials for genistein and the genistein copper(II)-complex. In contrast, for DPPH^•^, hydrogen atom transfer [27,32] may be dominate and at a slower rate.

Genistein increases the radical scavenging efficiency upon coordination to copper(II). An efficient binding of genistein to copper(II), however, depends on the polarity of the solvent and is favored by less polar solvents. The important coordination of flavonoids like genistein for increasing the antioxidant efficiency through improved radical scavenging may accordingly be most significant in interfaces between aqueous media and lipid layers, such as in membranes. The increased radical scavenging efficiency is further concluded to result from perturbation of the conjugated bonding system of genistein rather than from formation of copper(I) by an internal electron transfer from the genistein ligand to the metal center.

## 4. Materials and Methods

### 4.1. Chemicals and Reagents

Genistein (Gen) and daidzein (Daid) were from Huike Plant Exploitation Inc., (>98%, for both compounds, Shanxi, China) and used as received. Cu(II)SO_4_·5H_2_O from Beijing Chemical Works (>99%, Beijing, China) was used as received. All-*trans*-*β*-carotene (*β*-Car) was from Sigma-Aldrich (St. Louis, MO, USA), and the *β*-Car was purified by recrystallization in *n*-hexane/acetone mixed solutions, and the purity checked by high-performance liquid chromatography (HPLC) was ~98%. 1,1-Diphenyl-2-picrylhydrazyl radical (DPPH^•^) was purchased from ZhongShengRuitai Technology Inc. (>97%, Beijing, China). Ferrocene (98%) and NaClO_4_ (>98%) were from Sigma-Aldrich (St. Louis, MO, USA).

HPLC grade methanol (Mreda Technology, Inc., Lake Forest, CA, USA) and spectral purity grade ethanol (≥99.9%, Fine Chemical Industry Research Institute, Tianjin, China) were used as received. Ultrapurified water was prepared by Mingche ^TM^-D 24^UV^ (Merck Millipore Corp., Shanghai, China). Tetrahydrofuran (THF, >99.8%, Feng Yue Chemical Inc., Tianjin, China) was HPLC-grade. Chloroform (>99.0%, Beijing Chemical Works, Beijing, China) was purified before use by passing through an alumina column (AR, Tianjin Fuchen Chemical Plant, Tianjin, China). Solutions of the genistein dianion were prepared by addition of 2 equivalents of tetramethylammonium hydroxide (CH_3_)_4_N^+^OH^−^ (97%, Sigma Aldrich, St. Louis, MO, USA) to a neutral solution [38]. Methanol-*d*_4_ and chloroform-d (>99.8%) were purchased from Sigma-Aldrich (St. Louis, MO, USA).

### 4.2. Reaction of Cu(II) with Genistein

All UV−vis absorption spectra were measured on a Cary50 spectrophotometer (Varian, Inc., Palo Alto, CA, USA) using 1.0 cm quartz cells in a thermostated room of 25 °C. The solutions were prepared by mixing solutions of genistein and copper(II) with total molar concentrations of 1.0 × 10^−4^ M in ratios varying from 1:9 to 9:1, and Job’s plots were obtained from the absorbance at 410 nm versus mole fraction of copper(II) for experiments in methanol/chloroform (7/3, *v*/*v*), methanol, and ethanol. For alkaline methanol/chloroform (7/3, *v*/*v*), 425 nm absorbance was used for the calculations. Reactions of copper(II) with daidzein were used for comparison in methanol/chloroform (7/3, *v*/*v*).

### 4.3. NMR and Mass Spectra Characterization

^1^H-NMR spectra were obtained on a Bruker AM 400 MHz spectrometer (Karlsruhe, Germany). MS spectra were obtained on a Thermo Scientific™ Q Exactive™ HF (Waltham, MA, USA) in the positive ion mode. The samples were prepared by mixing solutions of genistein and copper(II), and then the mixed solutions were pushed through the nylon membrane with 220 nm sieve pores. The samples were analyzed by direct infusion ESI by means of a syringe pump (Thermo UltiMate 3000, Waltham, MA, USA) at a flow rate of 5 μL/min. Capillary temperature is 320 °C and spray voltage is 3.50 kV.

### 4.4. Determination of Oxidation Potentials

Cyclic voltammetry (CV) was performed on a three-electrode CHI 760D electrochemical analyzer (ChenHua Instruments Inc., Shanghai, China). The working electrode was a glassy carbon piece (diameter = 4 mm), the reference electrode was a silver wire [39], and the auxiliary electrode was a platinum wire. 0.10 M NaClO_4_ was used as supporting electrolyte. 5.0 × 10^−5^ M ferrocene was used as internal standard and CVs were obtained in the potential range of −0.5 V to +1.4 V at 0.1 V/s scan rate. The concentration of genistein and copper(II) were 1.0 × 10^−4^ M and 5.0 × 10^−5^ M, respectively.

### 4.5. β-Carotene Bleaching Assay and β-Carotene Radical Cation Quenching Kinetics

The laser pulses at 532 nm (4 mJ/pulse, 7 ns, and 10 Hz) for the bleaching assay were supplied by a Nd^3+^: YAG laser (Quanta-Ray PRO-230, Spectra Physics Lasers, Inc., Mountain View, CA, USA). The samples in the bleaching assay were irradiated by pulse laser for 3 minutes. The laser flash photolysis apparatus for quenching kinetics was described in detail in reference [40]. The same excitation laser pulses at 532 nm (3 mJ/pulse) as for the bleaching assay were used. 510 and 940 nm probe lights were provided by a laser-driven white light source (LDLS-EQ-1500, Energetiq Technology, Inc., Woburn, MA, USA). The kinetics were detected with a photodiode (model S3071, Hamamatsu Photonics, Hamamatsu, Japan). The optical path length of the flow cuvette was 5 mm. All of the measurements were carried out in a thermostated room of 25 °C. Methanol/chloroform (7/3, *v*/*v*) was used as the solvent.

### 4.6. DPPH Radical Scavenging

Absorption spectra of DPPH^•^ at different delay times were recorded in the absence and in the presence of genistein, CuSO_4_, and for mixtures of genistein and CuSO_4_. Decrease of absorbance at 521 nm with time was used for determining the radical-scavenging activities. Methanol/chloroform (7/3, *v*/*v*) was used as the solvent.

## Figures and Tables

**Table 1:** Coordination ratios (Cu(II):Gen) and stability constants (K_C_) for complexes of copper(II) and genistein (Gen), as formed in methanol, ethanol, and methanol/chloroform (7/3, *v*/*v*) in the absence and presence of (CH_3_)_4_N^+^OH^−^. Polarity (P(ε)) of solvents including H_2_O and THF with no complex formation.

Solvent	H_2_O	CH_3_OH	C_2_H_5_OH	CH_3_OH:CHCl_3_ = 7/3	THF
Cu(II):Gen	---/---	^a^ 1:1/^b^ 1:1	^a^ 1:2/^b^ 1:2	^a^ 1:2/^b^ 1:1	---
K_C_	---/---	^a^ 6.0 × 10^5^/^b^ 8.8 × 10^4^	^a^ 1.1 × 10^11^/^b^ 4.1 × 10^8^	^a^ 1.1 × 10^11^/^b^ 9.8 × 10^4^	---
^c^P(ε)	0.963	0.914	0.887	0.887	0.684

--- no complex formation detected; ^a^ neutral; ^b^ solvent added 2 equivalent of (CH_3_)_4_N^+^OH^−^ compared to genistein; ^c^P(ε): calculated according to $P\left(\varepsilon \right)=\frac{\varepsilon -1}{\varepsilon +2}$; where ε is dielectric constant, and P is the polarity of solvents calculated from reference [28]. ε of methanol/chloroform (7/3, *v*/*v*) is calculated according to reference [29].

**Table 2:** ESI mass spectra ions peaks (*m*/*z*) of Cu(II)Gen_2_ and Cu(II)Gen complexes formed in methanol:chloroform (7/3, *v*/*v*) and methanol.

Methanol:Chloroform = 7/3			Methanol		
*m*/*z* Experimental	*m*/*z* Calculated	Cu(II)Gen_2_	*m*/*z* Experimental	*m*/*z* Calculated	Cu(II)Gen
271.0594	271.0607	Gen + H^+^	271.0593	271.0607	Gen + H^+^
349.1825	349.0090	(Gen‒H) + Na^+^ + K^+^ + H_2_O	327.2522	327.0271	Gen + K^+^ + H_2_O
393.2088	393.1397	Gen + 5H_2_O + CH_3_OH + H^+^	396.0254	396.0370	(Gen‒H) + ^63^Cu^2+^ + 2CH_3_OH
437.2352	437.1241	Gen + 4CH_3_OH + K^+^	398.2316	398.0252	(Gen‒H) + ^65^Cu^2+^ + 2CH_3_OH
481.2611	481.2285	Gen + H_2_O + 6CH_3_OH + H^+^	488.3938	488.9968	Gen‒2H + 4H_2_O + CH_3_OH + 3K^+^
525.2871	525.2159	Gen + 4H_2_O + 5CH_3_OH + Na^+^	516.4255	516.0967	Gen‒H + 4H_2_O + 3CH_3_OH + 2K^+^
569.3133	569.2212	Gen + 2H_2_O + 7CH_3_OH + K^+^	544.4503	544.2731	Gen + 8CH_3_OH + H_2_O + H^+^
602.0257	602.0374	2(Gen‒H) + ^63^Cu^2+^ + H^+^			
604.0252	604.0256	2(Gen‒H) + ^65^Cu^2+^ + H^+^			
701.4918	701.2446	2Gen + 5CH_3_OH + H^+^			

**Table 3:** Chemical shifts (ppm) of genistein (Gen) and Cu(II)Gen_2_ complex in deuterated methanol/chloroform (7/3, *v*/*v*).

Chemical Shift/ppm	2-H	6-H	8-H	2’,6’-H	3’,5’-H
Gen	8.0	6.2	6.4	7.4	6.9
Cu(II)Gen_2_	8.2	6.3	6.5	7.5	6.8
